# The Perfect Storm: Coronavirus (Covid-19) Pandemic Meets Overfat Pandemic

**DOI:** 10.3389/fpubh.2020.00135

**Published:** 2020-04-23

**Authors:** Philip B. Maffetone, Paul B. Laursen

**Affiliations:** ^1^Independent Researcher, Ormond Beach, FL, United States; ^2^Auckland University of Technology, Auckland, New Zealand

**Keywords:** Covid-19, infectious disease, chronic disease, obesity, pandemic, immunity, lifestyle

## Introduction

In December, 2019, China's Wuhan city became the center of an outbreak of pneumonia of unknown cause, and by January, Chinese scientists reported to have isolated a novel coronavirus, severe acute respiratory syndrome coronavirus 2 (SARS-CoV-2; previously known as 2019-nCoV), from the infected patients (1, 2). The virus was later designated coronavirus disease 2019 (COVID-19) in February by the World Health Organization (3).

Other respiratory infectious pathogens, including strains of influenza virus type A and adenovirus (ADV), such as H1N1, H7N9, ADV 7, and ADV 55, often lead to worldwide outbreaks that seriously endanger human health. For example, by the end of 2009, the local H1N1 flu epidemic peaked in most countries with ~70,000 laboratory-confirmed hospitalized patients and over 2,500 fatal cases observed across 19 countries (4).

The earliest scientific data on Covid-19 from China shows those most vulnerable to infection have pre-existing illness that includes diabetes, hypertension, cardiovascular disease, and chronic inflammation (1, 5, 6). Many of these conditions are caused by excess body fat; a condition termed *overfat* (7, 8). The overfat condition itself is a significant yet little discussed risk factor in infectious viral diseases (9, 10), with overfat negatively affecting immune function and host defense mechanisms (11). It has been shown that both viral and bacterial pathogenesis is adversely altered in overfat hosts (11–14). While the viral infections all have different responses in human hosts, albeit similar, hospitals, and other critical care centers are applying their knowledge and skills concerning influenza to those with Covid-19 until more data and research is available specific to the Covid-19 virus (15).

Some viruses have high intrinsic levels of pathogenicity, mediating significant tissue damage in larger numbers of infected individuals, including smallpox and Ebola, with increased risk of death (16). While the Covid-19 produces symptoms common in other viral infections (such as fever, dry cough, dyspnea), it targets the lower airways to increase respiratory tissue damage, producing significantly high levels of plasma pro-inflammatory cytokines (17). In addition, some unique clinical features include upper respiratory tract symptoms like rhinorrhoea, sneezing, and sore throat, intestinal symptoms like diarrhea, and tissue infiltration of the upper lobe of the lung (17). Covid-19 also targets the central nervous system (18). It may be too early to know whether Covid-19 is capable of immune evasion (the blunting of an effective immune response) associated with increased tissue damage, especially in those with impaired immunity (16).

Currently, there are no specific or effective antiviral drugs, nor vaccines against COVID-19 infection, for potential therapy of humans (17). This makes prevention through healthier lifestyle an important underutilized and significant preventive measure. While extensive measures to reduce person-to-person transmission of COVID-19, like other infectious agents, are required to control the current outbreak, important preventive measures associated with lifestyle can help reduce the risks of future outbreaks.

The early death cases of COVID-19 were shown to occur primarily in elderly people who often have poor immune function that permits faster progression of viral infection (19). While most viral pandemics have similarities despite different pathogens, most hospitalizations occur among persons <2 years of age or 65 years of age or older, and among patients with certain medical conditions. One exception was during the 2009 pandemic influenza A (H1N1), which spread globally, with smaller numbers of severe illnesses reported among persons 65 years of age or older (~5%) (20).

While children have yet to develop full natural immunity, the elderly may have impaired immune responses. However, age may not be a single susceptibility, as many older individuals are physiologically more functional, possessing healthier lifestyles that include healthy eating and physical activities, in addition to potential genetic benefits (21).

The Covid-19 pandemic is spreading rapidly throughout the world, with few effective tools to help treat those who are sick. Current treatment strategies are limited to quarantine, isolation, and implementation of infection-control measures to prevent spread (22).

As of March 31, 2020, 750,890 cases and 36,405 deaths due to coronavirus disease 2019 (COVID-19), caused by the novel severe acute respiratory syndrome coronavirus 2 (SARS-CoV-2), had been reported worldwide (23).

As with reports out of China and Italy, data from the U.S. demonstrates those at higher risk for Covid-19 had chronic conditions, with 78% of COVID-19 patients requiring admission to the intensive care unit (ICU) and 94% of hospitalized patients who died had an underlying condition (24). Underlying conditions included diabetes, cardiovascular disease, chronic lung disease (including asthma, chronic obstructive pulmonary disease, and emphysema), hypertension, and cancer (1, 5, 6). Most of these underlying conditions are caused by or are associated with excess body fat (8, 25).

While those ≥65 years of age were more at risk, those admitted to the ICU in the age bracket of 19–64 years also had significantly more chronic illness than those hospitalized without ICU admission (23, 24). These conditions are primarily caused by excess body fat and its associated chronic inflammation (8, 25). These and other analyses may be limited by relatively small numbers, missing data due to the burden placed on reporting health departments, and the rapidly rising number of cases (23, 24).

Initial indications in the U.S. showed that fatality was highest in persons aged ≥65 years, 1–3% among persons aged 55–64 years, <1% among persons aged 20–54 years, while no fatalities occurred among persons aged ≤ 19 years (26). Worldwide, mortality is expected to vary with the underlying chronic illness, with the risks associated with COVID-19 heavily influenced by the presence of these comorbidities (1, 5, 6, 27).

## The Hidden Overfat Pandemic

The overfat pandemic and its associated chronic inflammation and insulin resistance, and downstream chronic disease represents one of the greatest threats to global human health (28). Excess body fat is a primary driver of chronic inflammation, insulin resistance, and many downstream chronic illnesses, including cardiovascular disease, diabetes, hypertension, liver and kidney disease, cancer, and others (7, 8, 29), including increased risk of respiratory infections and inflammatory lung diseases (30).

During and after the 2009 influenza A/H1N1 pandemic, body mass index (BMI) was recognized as an independent risk factor for influenza, in particular, the severity of the illness, hospitalization, increased risk of spreading the disease, and death (9, 10). Data from past pandemics and seasonal influenza demonstrate that obesity is an independent risk factor for severe outcomes (10, 31).

**Figure 1:**
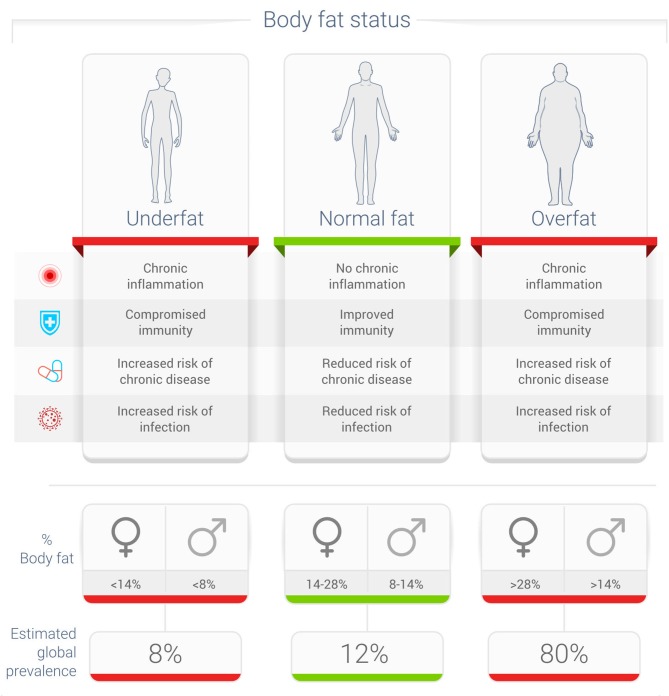
Potential relationship between body fat status and rates of infection (7, 8, 29).

Unfortunately, most metrics used in studies of influenza and other viral infections use *obesity* as a metric, and not *adiposity*, which may be a better metric to define this relationship between excess body fat and influenza (31). Even more important is the fact that 40 percent or more of normal-weight non-obese adults may have excess body fat that impairs their health—the condition called overfat (Figure 1) (7).

## Overfat and Impaired Immunity

Adipose tissue is a multifunctional endocrine organ involved in many physiological and metabolic processes, and is also populated by a number of immune cells including T lymphocytes and macrophages (32, 33). Excess body fat, however, can impair immunity, with obese individuals having a higher incidence of immune and autoimmune diseases (28). Excess body fat can contribute to cardiovascular and metabolic health impairment including various risk factors such as abnormal blood glucose, high-density lipoprotein cholesterol (HDL), triglycerides, and blood pressure, which progress to a variety of diseases including type 2 diabetes, non-alcoholic fatty liver, cancers, Alzheimer's, and cardiovascular diseases (34–36).

**Figure 2:**
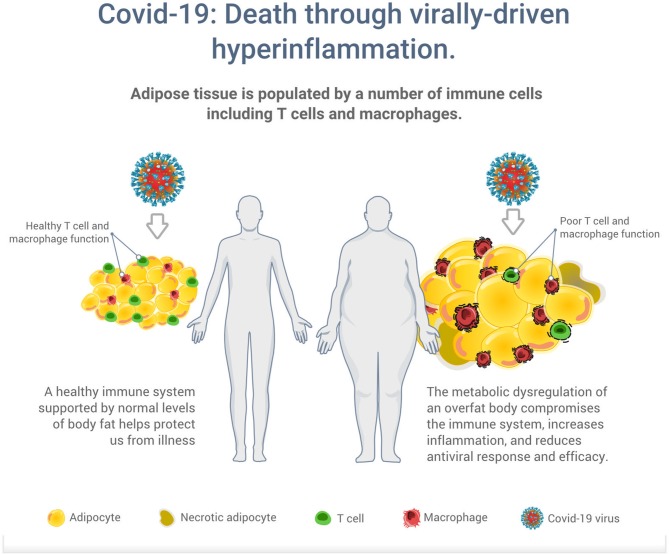
Illustration of the potential increased risk of death through virally driven hyperinflammation in overfat hosts. Excess adipose tissue promotes systemic inflammation and is characterized by infiltration and activation of immune cells secreting pro-inflammatory mediators, such as cytokines, adipokines, and chemokines, which secrete additional pro-inflammatory molecules. In addition to T cells and macrophages, these immune cells also include neutrophils, B1 and B2 cells, NK cells, and innate lymphoid cells (11).

While humans are constantly infected with multiple endogenous and exogenous viral agents, with an estimated generation of up to 10^12^ new virus particles per day, a healthy immune system protects us in most situations from illness (37). However, the metabolic dysregulation of an overfat body can compromise the immune system to increase the risk of infections, and chronic respiratory diseases (38, 39). Overfat has also been shown to aggravate the effect of seasonal influenza on respiratory mortality independent of the effect of comorbidities and meteorological factors (31). As illustrated in Figure 2, excess body fat has been proposed as a driver of poor T cell and macrophage function, reduced antiviral responses and efficacy, increased viral shedding and subsequent transmission (12, 32, 33, 40, 41). While vaccines have been the hallmark of primary preventive measures against many infections, it appears vaccines also work less effectively in an overfat body (42).

Overfat hosts also may have a breakdown of the respiratory epithelium leading to fluid influx in the airway space (43), with obese mice more likely than lean mice to have increased lung permeability during infection (12). The increased incidence of complications in hospitalized obese patients with influenza infections may be due to increased viral spread to other respiratory areas, further reducing lung function and increasing mortality (44). Overfat is also associated with impaired or reduced fat oxidation rates, which is a hallmark of aging and disease (45).

## Other Lifestyle Factors

Food consumption is a major factor influencing body fat content, the immune system, overall health and the risk of developing diseases (32). The intake of dietary sugar and other refined carbohydrates plays a primary role in the overfat pandemic (29). Importantly, very-low carbohydrate/ketogenic diets have been successfully applied in conditions that include epilepsy, metabolic disorders, cancer, neuronal loss, and muscle and nerve degeneration (46, 47). The diets have also been successful in reducing excess body fat (48) and chronic inflammation (49). Very-low carbohydrate/ketogenic diets may also be protective in promoting a positive immune response against influenza virus infection (50).

While infection rates are still evident in warm weather environments, optimal immune function is also dependent upon a variety of nutritional factors, in addition to regular sunlight exposure to increase vitamin D levels (51). Vitamin D can act as an immune modulator, prevent excessive expression of inflammatory cytokines, increase the “oxidative burst” potential of macrophages, stimulate the expression of anti-microbial peptides in neutrophils, monocytes, natural killer cells, and in epithelial cells lining the respiratory tract where they play a protective role (52). However, those who are overfat have consistently lower vitamin D levels across age, ethnicity, and geography (53). The seasonality of infectious disease outbreaks suggests that environmental conditions have a significant effect on disease risk. In particular, ultraviolet radiation from sun exposure and associated increases in vitamin D levels share common pathways of innate immune activation (54).

## Conclusion

While we await more data on Covid-19, comorbidity risk factors that are associated with overfat appear related (1, 5, 6, 27). The Covid-19 and overfat pandemics are two serious public health concerns that are correlated, despite having very different horizons and timescales. Both require urgent attention. Jones (55) writes in the New England Journal of Medicine that, while some experts warn half the world's population could be infected by the end of 2020, resulting in more than 100 million deaths, such a perfect storm is exceedingly rare. It is however, regrettably, one that is possible. Perhaps a more important lesson for the world may be that we control much of our health, and that the prevention of infections through a healthy immune system is, not unlike chronic disease and physical impairment, strongly associated with a healthy lifestyle.

## Author Contributions

PL and PM conceived the idea for the manuscript through discussion and observation of the COVID-19 pandemic. The manuscript was lead by PM with contributions made by PL.

## Conflict of Interest

PM is part owner of a business that promotes health and wellness (MAFF PTY). PL is part owner of a business that promotes health and wellness (HIIT Science Inc).
